# Added value of ^68^Ga-PSMA PET/CT for the detection of bone metastases in patients with newly diagnosed prostate cancer and a previous ^99m^Tc bone scintigraphy

**DOI:** 10.1186/s13550-020-00618-0

**Published:** 2020-04-08

**Authors:** Helle D. Zacho, Søren Ravn, Ali Afshar-Oromieh, Joan Fledelius, June A. Ejlersen, Lars J. Petersen

**Affiliations:** 1 Department of Nuclear Medicine and Clinical Cancer Research Center, Aalborg University Hospital, Hobrovej 18-22, Postboks 365, DK-9100 Aalborg, Denmark; 2 Department of Clinical Medicine, Aalborg University, Aalborg, Denmark; 3 Department of Nuclear Medicine, Bern University Hospital, University of Bern, Bern, Switzerland; 4 Department of Nuclear Medicine, Regional Hospital West Jutland, Herning, Denmark

**Keywords:** PSMA PET/CT, Bone scintigraphy, Bone scan, Bone metastases, Newly diagnosed prostate cancer

## Abstract

**Purpose:**

To investigate the added value and diagnostic accuracy of ^68^Ga-PSMA PET/CT versus bone scintigraphy (BS) for bone metastasis detection at the primary staging of prostate cancer (PCa).

**Methods:**

Inclusion criteria involved consecutive patients with newly diagnosed intermediate- to high-risk PCa, who had undergone BS, mostly with supplementary SPECT/low-dose CT, and ^68^Ga-PSMA-11 PET/CT within less than 3 months without therapy initiation between the two investigations. BS was evaluated according to clinical routine and reported as no bone metastases (M0), bone metastases (M1), or equivocal (Me). The ^68^Ga-PSMA-11 PET/CT was blindly evaluated by three specialists as M0, M1, or Me at the patient level. Sensitivity analyses were conducted using a “best valuable comparator” using all available imaging and clinical follow-up as a reference.

**Results:**

In total, 112 patients were included; ^68^Ga-PSMA-11 PET/CT showed a sensitivity of 1.00, specificity of 0.93–0.96, positive predictive value of 0.74–0.81, and negative predictive value of 1.00. ^68^Ga-PSMA-11 PET/CT revealed bone metastases in 8 of 81 patients with M0 disease according to BS. ^68^Ga-PSMA-11 PET/CT confirmed the presence of bone metastases in all patients (*n* = 9) with M1 disease according to BS. In patients with Me by BS, ^68^Ga-PSMA PET/CT provided a definite result in 20 of 22 patients. ^68^Ga-PSMA-11 PET/CT resulted in a false-positive answer in four patients with solitary rib lesions.

**Conclusion:**

^68^Ga-PSMA-11 PET/CT revealed bone metastases in 10% of patients without bone metastases on BS and in 36% patients with indeterminate BS. However, solitary PSMA-avid lesions in the ribs should be interpreted cautiously as they may represent false-positive findings.

## Introduction

According to the European Association of Urology (EAU) guidelines, bone scintigraphy (BS) is the recommended imaging modality for the detection of bone metastases in patients with newly diagnosed unfavorable intermediate- to high-risk prostate cancer (PCa) [1]. The presence of bone metastases as well as the number of bone metastases are of great significance to tailor the treatment [1–3] and to determine patients’ prognosis [4].

Positron emission tomography/computed tomography with ligands of the prostate-specific membrane antigen (PSMA PET/CT), especially with the ^68^Ga-labelled ligand PSMA-11, has been used widely to assess PCa metastases, particularly at the time of biochemical recurrence [5, 6], but ^68^Ga-PSMA PET/CT has also been shown to possess premium diagnostic accuracy for the detection of bone metastases at the time of primary staging [7, 8].

The purpose of this study was twofold. First, we aimed to evaluate the added value of ^68^Ga-PSMA-11 PET/CT in patients with newly diagnosed PCa versus the value of the initial BS and to assess the diagnostic accuracy of ^68^Ga-PSMA-11 PET/CT for skeletal metastasis patients with PCa at the time of primary staging.

## Materials and methods

### Patients

From May 2015 to October 2018, all patients undergoing PSMA PET/CT at our department were screened for inclusion in the present retrospective study. The eligibility criteria were as follows: (1) newly diagnosed with PCa, (2) no prior treatment for PCa, (3) ^99m^Tc BS performed within 3 months of the ^68^Ga-PSMA-11 PET/CT, and (4) no treatment for PCa initiated between BS and PSMA PET/CT. All patients had BS and a CT scan of the thorax, abdomen, and pelvis conducted as part of the routine staging procedure according to the recommendations of the EAU [1].

### ^68^PSMA-PET/CT

In short, the ^68^Ga-PSMA-11 ligand was used in the present study and was synthesized as previously described [9]. The ^68^Ga-PSMA-11 PET/CT was performed in accordance with the guidelines of the European Association of Nuclear Medicine/Society of Nuclear Medicine (EANM) on ^68^Ga-PSMA-11 PET/CT [10]. Images were acquired approximately 60 min after an intravenous injection of 2 MBq/kg body weight (minimum 100 MBq, maximum 200 MBq). Patients were examined using either a VCT Discovery True 64 PET/CT system (GE Healthcare, USA) or a Siemens Biograph mCT Flow 64 PET/CT system (Siemens, Erlangen Germany). The patients were scanned from the base of the skull to the upper thigh, and the PET images were acquired in 3D mode. The PET images were reconstructed using attenuation correction using an ordered subset expectation-maximization algorithm. For the Siemens Biograph, mCT Flow 64 PET/CT time-of-flight and point-spread-function were applied. A low-dose CT was performed immediately after the PET scan and used for attenuation correction and anatomical co-registration.

### ^99m^Tc bone scintigraphy

BS was conducted in accordance with the EANM guidelines for BS [11]. A planar whole body BS scan was acquired on a two-headed gamma camera (Symbia T16, Siemens Medical Solutions, Erlangen, Germany) 2–3 h after the intravenous injection of 9.4 MBq ^99m^Tc-labelled methylene bisphosphonate per kilogram body weight (minimum 750 MBq). A supplemental single-photon emission computed tomography (SPECT)/CT, covering one- or two-bed positions (each 40 cm field of view), was conducted at the discretion of the physician in charge. SPECT/CT was acquired using the following parameters: 16 views with 10 s per view, as previously described [12]. The images were reconstructed using iterative reconstruction with scatter correction. A low-dose CT was performed for attenuation correction and anatomical co-registration. Planar bone scan and any supplementary SPECT/low-dose CT was considered the standard bone evaluation per clinical guideline recommendation. According to institutional practice, a contrast-enhanced CT was performed after the bone scan for the assessment of lymph node and soft tissue metastasis. The information from the “bone window” was not included in the comparison with PSMA PET. In retrospect, no patients had any findings in the contrast-enhanced bone window, which changes the overall BS classification of metastasis.

### Observers and procedure for image assessment

The evaluation of BS was a part of the daily clinical practice. The BS results were evaluated by at least two observers, mostly one physician in training and re-read by at least one experienced board-certified specialist in nuclear medicine. The consensus result was categorized as either (1) no bone metastases, (2) equivocal for bone metastases in which case it was an institutional practice to conduct a ^68^Ga-PSMA-11 for confirming or ruling out bone metastases, or (3) bone metastases.

The ^68^Ga-PSMA-11 PET/CT images were evaluated by three board-certified nuclear medicine physicians with experience in the evaluation of ^68^Ga-PSMA-11 PET/CT [13]. The evaluation of ^68^Ga-PSMA-11 PET/CT was conducted according to Rauscher et al. [14]. The PSMA PET/CT images were categorized on a three-point-scale: bone metastases (M1), no bone metastases (M0), or equivocal in patients with bone lesions not typical for bone metastases (Me). Furthermore, the exact number and location of all equivocal or metastatic bone lesions were described in patients with 10 or fewer lesions. The observers had no access to any clinical information except that the patients were newly diagnosed with prostate cancer and were instructed to evaluate the PSMA PET using the corresponding CT-images as they would do in their daily clinical practice. Likewise, the observers were blinded to the evaluation of the BS. Cases of disagreement were resolved by consensus.

### Best valuable comparator

In most cases, a histopathologic reference standard was not available for ethical and practical reasons. For the present study, we defined a “best valuable comparator” (BVC) for the presence or absence of bone metastases at the patient level similar to previous diagnostic studies of bone metastases [6, 8, 15]. The BVC was based on all available imaging results available at the time of staging as well as supplementary and/or follow-up imaging (all modalities). A minimum of 12 months of clinical and laboratory follow-up was required. Patients with PSA < 0.1 ng/mL after radical prostatectomy without any systemic treatment were categorized as having no bone metastases at the time of staging [16].

### Statistics

Diagnostic accuracy was calculated among patients with a valid BVC. Sensitivity analyses were performed for each component of the diagnostic accuracy endpoints (sensitivity, specificity, positive, and negative predictive values), where patients with equivocal findings were first considered positive for metastases (pessimistic analyses) and then calculated as negative for metastases (optimistic analysis). The results are summarized by the mean or median values, standard deviations and ranges. Statistical analyses were performed using STATA®11 (StataCorp LP, College Station, TX, USA). All results are reported with 95% confidence intervals (CIs).

### Ethics

Due to the retrospective nature of the study, no formal approval from the ethics committee was required according to our national legislation. The study was approved by the Danish Data Protection Agency (approval number 2008-58-0028).

## Results

### Patients

One hundred eighteen consecutive patients underwent ^68^Ga-PSMA-11 PET/CT at the time of initial staging, and of these patients, 6 did not undergo BS within 3 months of the ^68^Ga-PSMA-11 PET/CT. Thus, 112 patients were included in the analysis. The great majority (*n* = 99, 88%) of the patients had high-risk disease according to the EAU criteria (Table 1). Twenty-two patients (20%) were referred for a ^68^Ga-PSMA-11 PET/CT solely due to equivocal results from initial staging BS, and 30 patients were referred due to equivocal lesions identified by CT (Table 1). The initial BS showed no metastases in 81 patients (72%), equivocal results in 22 patients (20%), and bone metastases in 9 patients (8%). BS was conducted prior to ^68^Ga-PSMA-11 PET/CT in all patients. A BVC at the patient level was available in 105 of 112 (94%) patients with a median follow-up time of 21 months (range 12–50 months). In seven patients, no firm conclusion regarding the presence or absence of bone metastases was achievable. This result was mainly due to patients undergoing androgen-deprivation therapy (ADT) without any follow-up imaging.

**Table 1:** Demographic data

Patient demographics (*n* = 112)		
Age (years), mean (range)	68	(48–78)
PSA (ng/mL), mean (range)	34.5	(1.7–276)
median	21	
Gleason		
7 (3 + 4), *n*	17	(15.2%)
7 (4 + 3), *n*	24	(21.4%)
8, *n*	10	(8.9%)
9, *n*	61	(54.5%)
T-stage		
T1, *n*	24	(21.4%)
T2, *n*	37	(33.0%)
T3, *n*	47	(42.0%)
T4, *n*	4	(3.6%)
EAU risk score		
Favorable* intermediate, *n*	7	(6.3%)
Unfavorable intermediate risk, *n*	6	(5.4%)
High risk, *n*	99	(88.4%)
Reason for PSMA-PET/CT		
CT equivocal	30	(26.8%)
Bone scan equivocal	22	(19.6%)
Very high risk and no metastases according to BS or CT	28	(25.0%)
Participation in a PSMA-study	20	(17.9%)
Miscellaneous	12	(10.7%)
Bone scintigraphy prior to inclusion		
Planar whole body	23	(20.5%)
Planar whole body + SPECT/CT	89	(79.5%)
Time between PSMA-PET/CT and BS (days)		
Median (range)	22	(6–80)

*BS* bone scintigraphy, *CT* computed tomography, *EAU* European Association of Urology, *SPECT* single-photon emission computed tomography

*Intermediate EAU risk differentiate between patients with Gleason 7 (3+4) as favorable and Gleason 7 (4 + 3) as unfavorable

### ^68^Ga-PSMA-11 PET/CT in patients classified as M0 by BS

**Fig. 1:**
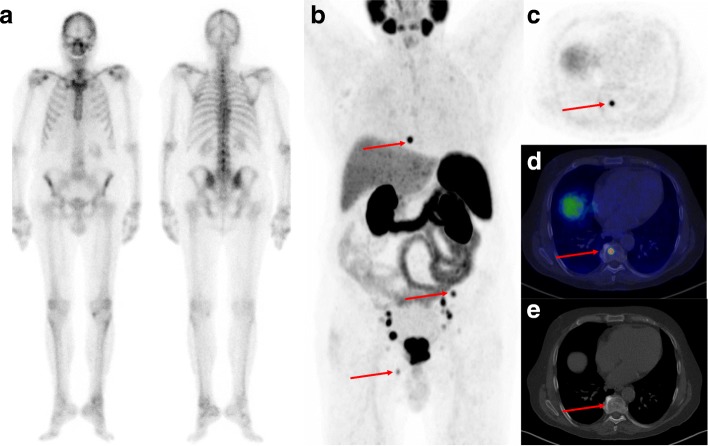
Example of a patient (PSA 44 ng/mL, Gleason score 9, T3) classified as M0 according to initial BS as shown in anterior (**a**) and posterior projection (**b**). The patient was referred for ^68^Ga-PSMA-11 PET/CT due to high-risk prostate cancer. The maximum intensity projection (MIP) of the ^68^Ga-PSMA-11 PET (**c**) revealed several lesions with avid ^68^Ga-PSMA-11 uptake, including three bone metastases marked with arrows (Th8, left iliac bone and right pubic bone). The axial ^68^Ga-PSMA-11 PET image of the lesion in Th8 is shown in **c** with a fused ^68^Ga-PSMA-11 PET/CT image shown in **d** and only a slight sclerotic change in the axial CT image (**e**). BVC confirmed M1 status

**Fig. 2:**
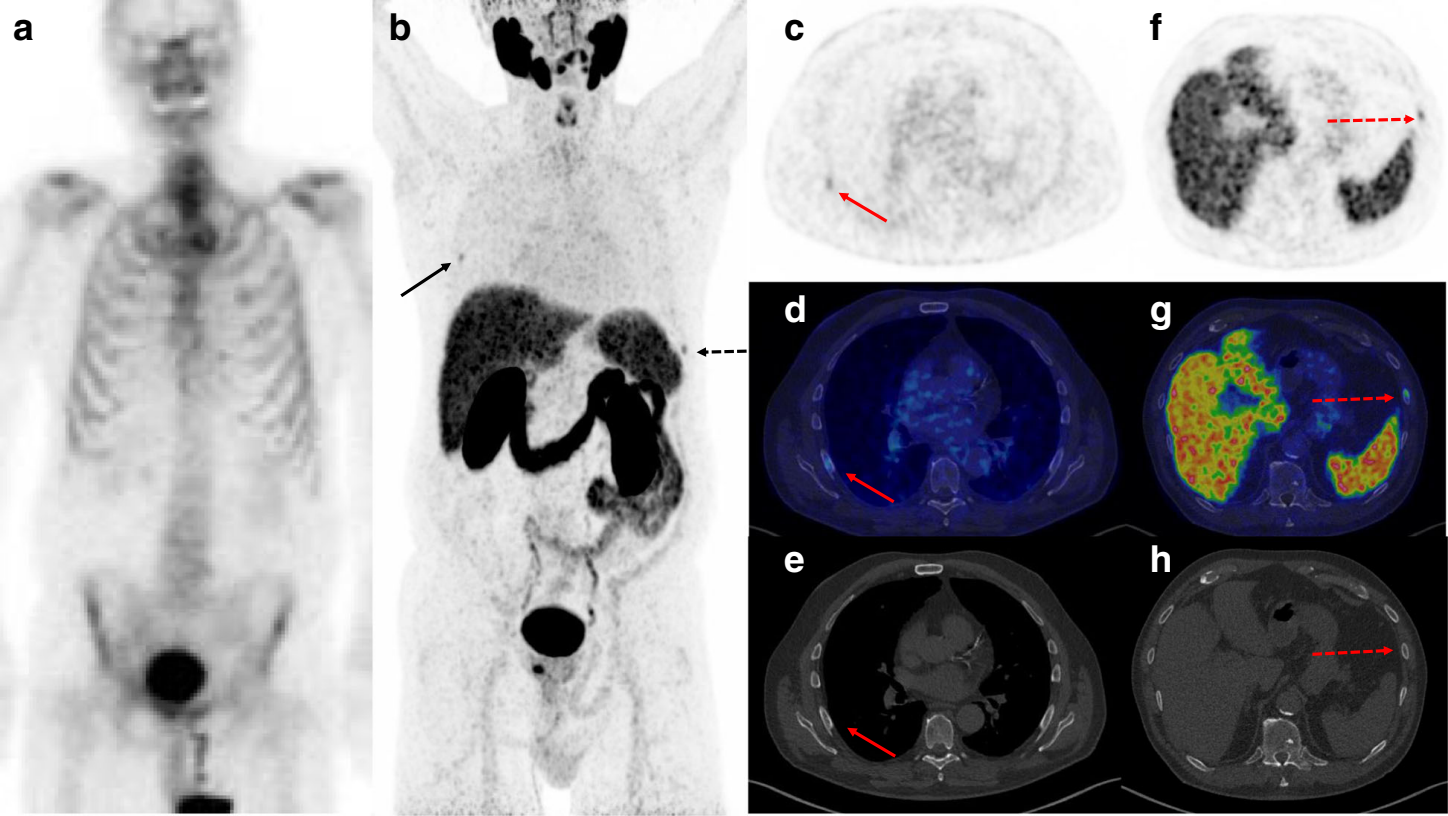
Example of a patient (PSA 13, Gleason score 9, T2b) with no bone metastases according to BS (shown in anterior projection) (**a**) but with two suspicious lesions in the seventh right rib and the eighth left rib according to the ^68^Ga-PSMA-11 PET/CT. Except for the ^68^Ga-PSMA-11 uptake in the ribs and the prostate, no other suspicious lesions were observed. The ^68^Ga-PSMA-11 PET MIP showed two rib lesions (**b**); the full arrow indicates a bone lesion in the seventh rib on the right side with corresponding axial ^68^Ga-PSMA-11 PET (**c**), ^68^Ga-PSMA-11 PET/CT fusion (**d**) and CT (**e**). The hatched arrow indicates a bone lesion in the left eighth rib with corresponding axial PSMA PET and corresponding axial ^68^Ga-PSMA-11 PET (**f**), ^68^Ga-PSMA-11 PET/CT fusion (**g**), and CT (**h**). The patient had no bone metastases according to the BVC: the patient had a radical prostatectomy without any systemic treatment, and the post-prostatectomy PSA level was < 0.1 ng/mL and remained so until this study was published

In patients without bone metastases based on BS (*n* = 81), ^68^Ga-PSMA-11 PET/CT showed metastasis-suspicious lesions in eight patients (10%) (Fig. 1) and equivocal results in two patients (2%). ^68^Ga-PSMA-11 PET/CT was negative for bone metastasis in 71 patients (88%). Among the eight patients with suspected bone metastases exclusively shown by ^68^Ga-PSMA-11 PET/CT, six had skeletal metastases confirmed by the BVC, whereas two patients had false-positive lesions (Fig. 2, Supplementary Table 1); both patients had PSMA-avid uptake in the ribs only. Two patients with equivocal findings by ^68^Ga-PSMA-11 PET/CT also presented with solitary PSMA uptake in the ribs, a BVC could not be established in both patients as they were referred for ADT and did not undergo follow-up imaging. ^68^Ga-PSMA-11 PET/CT did not show any false-negative cases among patients with a negative or equivocal ^68^Ga-PSMA-11 PET/CT.

### ^68^Ga-PSMA-11 PET/CT in patients with equivocal lesions on BS

**Fig. 3:**
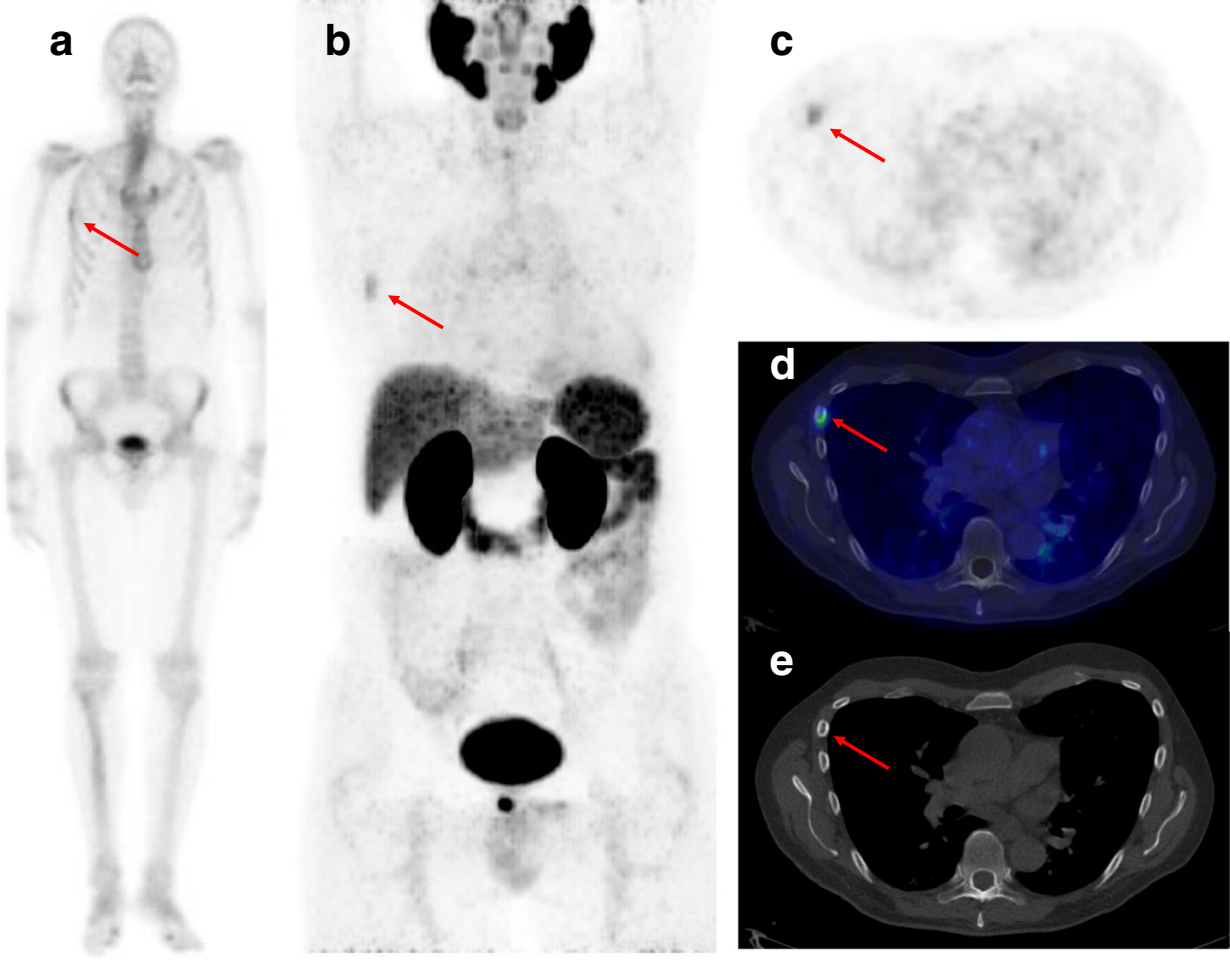
Example of a patient (PSA 6 ng/mL, Gleason score 7, T2b) with four equivocal lesions according to BS (shown in anterior projection) (**a**) including a lesion in the fourth right rib indicated by the arrow. According to the ^68^Ga-PSMA-11 PET/CT, the lesion was considered metastatic as shown by the ^68^Ga-PSMA-11 MIP (**b**) with the corresponding axial ^68^Ga-PSMA-11 PET (**c**), PET/CT fusion (**d**), and a slight sclerotic lesion in the axial CT image (**e**). Except for the high ^68^Ga-PSMA-11 uptake in the prostate, no other suspicious lesions were observed. The patient underwent a CT-guided biopsy that showed benign findings, and then the patient had a radical prostatectomy without any systemic treatment. The PSA dropped to < 0.1 ng/mL after prostatectomy and remained so for at least 12 months

In patients referred to ^68^Ga-PSMA-11 PET/CT due to equivocal lesions on BS (*n* = 22), ^68^Ga-PSMA-11 PET/CT provided a definitive diagnosis of bone metastases in 20 of 22 patients. PSMA-avid lesions suspicious for bone metastases were found in 9 patients (41%), ^68^Ga-PSMA-11 PET/CT was equivocal in 2 patients, and it was negative in 11 (50%) patients. Compared to the BVC, two of nine patients with PSMA-avid bone lesions had false-positive results based on ^68^Ga-PSMA-11 PET/CT (Supplementary Table 1). In both patients, the false-positive lesions were located in the ribs (Fig. 3).

### ^68^Ga-PSMA-11 PET/CT in patients classified as M1 by BS

**Fig. 4:**
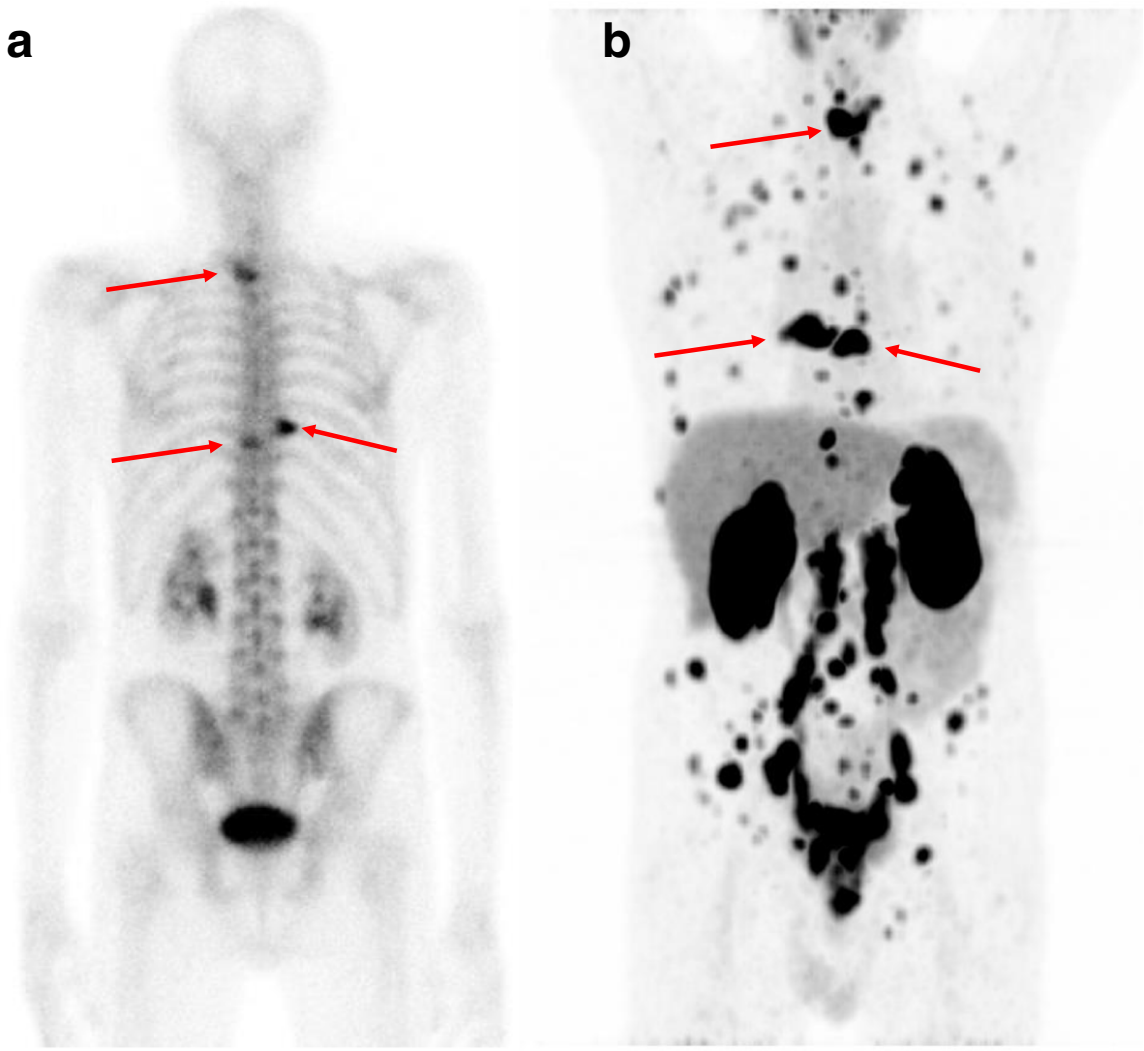
Example of a patient (PSA 8 ng/mL, Gleason score 9, T3) with three bone metastases (shown by the arrows) on BS (shown in posterior projection) (**a**), whereas the ^68^Ga-PSMA-11 PET/CT revealed numerous bone lesions and lymph nodes in the pelvis and abdomen. The MIP of the ^68^Ga-PSMA-11 PET/CT is shown in anterior projection (**b**), and the arrows indicate the bone metastases also shown by BS

In all nine patients with bone metastases according to BS, ^68^Ga-PSMA-11 PET/CT also showed PSMA-avid lesions suspicious of bone metastases. All patients were true positive for skeletal metastases according to the BVC. In one patient with three bone metastases according to the BS, ^68^Ga-PSMA-11 PET/CT revealed more than 10 lesions (Fig. 4). In three patients, ^68^Ga-PSMA-11 PET/CT increased the number of bone lesions identified from 4 to 5, 8, and 9. In the remaining five patients, the number and location of lesions identified by BS and ^68^Ga-PSMA-11 PET/CT were identical.

### Diagnostic accuracy of ^68^Ga-PSMA-11 PET/CT

Overall, the sensitivity of ^68^Ga-PSMA-11 PET/CT for the detection of bone metastases was excellent, with no false-negative patients (sensitivity 100%). The specificity ranged from 0.93 to 0.96, depending on whether equivocal findings were considered M0 or M1 per BVC (Table 2). Four patients had false-positive bone metastases according to ^68^Ga-PSMA-11 PET/CT, and one patient had equivocal lesions based on ^68^Ga-PSMA-11 PET/CT, which were benign according to the BVC. In all cases of false-positive lesions, the PSMA-avid lesions were located in the ribs and exhibited slight sclerotic, though unspecific, changes based on CT. Moreover, none of the patients with false-positive bone lesions had other PSMA-avid lesions, except for PSMA uptake in the prostate. One patient had a biopsy of the rib lesion (which was benign), and three patients underwent radical prostatectomy without any systemic treatment and their PSA levels remained below 0.1 ng/mL during follow-up for at least 12 months (Supplementary Table 1). A total of seven patients exhibited solitary PSMA uptake in rib lesions. The BVC did not declare any of these lesions as bone metastases; the BVC was M0 in five patients and a firm diagnosis of bone metastases could not be obtained in two of the patients. Thus, there was no cases of true positive solitary rib lesions on PSMA PET.

**Table 2:** Diagnostic accuracy of 68Ga-PSMA PET/CT among 105 patients with a final diagnosis (BVC) for the presence or absence of bone metastases

	M0 by BVC, *n* = 88			M1 by BVC, *n* = 17						
	M0	Equivocal	M1	M0	Equivocal	M1	Sensitivity (95% CI)	Specificity (95% CI)	PPV (95% CI)	NPV (95% CI)
Three-point scale by three observers	82	2	4	0	0	17	–	–	–	–
Optimistic analysis: Equivocal result considered M0	84		4	0		17	1.00 (0.81–1.00)	0.96 (0.89–0.99)	0.81 (0.58–0.95)	1.00 (0.96–1.00)
Pessimistic analysis: equivocal result considered M1	82		6	0		17	1.00 (0.81–1.00)	0.93 (0.86–0.98)	0.74 (0.52–0.90)	1.00 (0.96–1.00)

*BVC* best valuable comparator, *CI* confidence interval, *M0* no bone metastases, *Equivocal* findings are equivocal for bone metastases, *M1* bone metastases present, *PPV* positive predictive value, *NPV* negative predictive value

## Discussion

The present study investigated the added value of ^68^Ga-PSMA-11 PET/CT in patients with newly diagnosed PCa who recently underwent BS. ^68^Ga-PSMA-11 PET/CT diagnosed bone metastases in 10% of patients with negative BS results, provided a firm diagnosis in 20 of 22 patients referred for ^68^Ga-PSMA-11 PET/CT due to equivocal BS results, and confirmed bone metastatic disease in all patients with positive BS results, but ^68^Ga-PSMA-11 PET/CT also identified a notable proportion of patients in whom PSMA-avid lesions in the ribs were false positive.

Pyka et al. published the first comparison of ^68^Ga-PSMA-11 PET/CT with BS in patients with PCa, including 37 patients at primary staging [8]. They found a sensitivity and specificity of ^68^Ga-PSMA-11 PET/CT of 100% at primary staging. Likewise, Lengana et al. reported a sensitivity of 96% and specificity of 100% of ^68^Ga-PSMA PET/CT for the detection of bone metastases at primary staging [7]. In the present study, the sensitivity was comparable to that found in prior studies, whereas the specificity was slightly lower than the previously reported specificity, which might be explained by the four patients with false-positive PSMA-avid uptake in the ribs.

^68^Ga-PSMA-11 PET/CT has previously shown an ability to detect bone metastases in a proportion of patients without bone metastases apparent on BS. Lengana et al. reported that PSMA PET/CT revealed bone metastases in 8.4% of patients with negative BS results [7], which is in line with the present findings in which 10% of the patients without bone metastases on BS were considered metastatic by PSMA. Among patients with bone metastases on BS, PSMA PET/CT confirmed M1 bone disease in all patients and showed more metastasis-suspected lesions than those detected by BS. These findings are in line with recent observations [7].

Although PSMA PET/CT was indeterminate in some cases, it provided a definitive imaging diagnosis among 96.4% of the patients in this population at the primary staging. These findings are comparable to findings in patients with biochemical recurrence after curatively intended treatment, where PSMA has been shown to provide a definite diagnosis in 99% of patients [17].

The fact that four patients obtained false-positive results based on PSMA PET/CT was unexpected and has not been reported in prior studies [8, 17]. In each of these four patients presenting with a total of five PSMA-avid lesions of non-prostatic origin, morphologic changes were observed in the corresponding CT images. However, biopsy ruled out bone metastases in one patient. In addition, the incorporation of a PSA < 0.1 ng/mL 12 months after radical prostatectomy served as a relatively reliable verification of non-metastatic diseases in three patients. In accordance with the BVC, PSA-negative metastatic PCa in the mentioned patients is very unlikely. The false-positive findings were likely not due to reader inexperience; two of the observers (HDZ and AAO) were highly experienced and evaluated PSMA PET/CT in numerous trials [5, 6, 17]. After we performed the blinded evaluation of the ^68^Ga-PSMA-11 PET/CT in the present study, a number of cases with PSMA-avid bone uptake in benign skeletal conditions were published [18–21], revealing that fibrous dysplasia in the ribs or rib fractures may be PSMA-avid. In summary, these findings emphasize the need for careful interpretation of PSMA PET/CT in rib lesions.

The strength of the present study is the consecutive inclusion of patients. Due to the unique national security number in Denmark, it was possible to perform a thorough follow-up on patients included in the study, even if the patients moved across regions within the country. In the present study, detailed follow-up data were available in most patients. A shortcoming is that histologic confirmation is rarely available in imaging studies of the bone. However, performing biopsy routinely is not ethically reasonable, and consequently, a composite endpoint (BVC) was applied in the present study. A BVC has previously been used in diagnostic studies of bone metastases, including studies comparing ^68^Ga-PSMA-11 PET/CT to BS [6–8, 15]. One of the limitations of the BVC is that the index test itself often plays a key role in the definition of the BVC, as the interpretation of a new and promising method might be unreasonably trusted in the final conclusion based on the BVC. In addition, the extent of clinical, imaging, and biochemical data available for the BVC have seldom been reported. Here, we included detailed information for a minimum of 12 months for all patients with at least one lesion according to either BS or PSMA. A minority of patients did not have data allowing for a definitive conclusion of BVC. For these reasons, it is imperative to consider the intrinsic verification bias when using a BVC. For the present study, the limitations are reflected in the sensitivity of 1.00, which is likely to be overestimated.

Patients were excluded if they had received any kind of treatment for PCa prior to BS and ^68^Ga-PSMA-11 PET/CT, which indicates that the scans were not influenced by the negative effects of ADT on PSMA PET/CT as described previously [22].

Despite the consecutive inclusion of patients, the present population was biased because more than 20% of the patients were referred to ^68^Ga-PSMA-11 PET/CT due to equivocal findings in BS. The study was not a head-to-head comparative diagnostic test accuracy study. Therefore, no comparative analysis of diagnostic accuracy for the detection of bone metastases by BS was conducted. Likewise, the high proportion of patients with equivocal BS does not reflect the true frequency of equivocal findings in BS when SPECT/CT is applied, which in unselected populations has been shown to be approximately 10% at the time of initial staging [23]. In the present setting, approximately 80% of the patients had a SPECT/CT performed as an add-on to the planar bone scintigraphy which is a limitation of the study. However, the use of SPECT/CT in patients with planar BS with possible benign or equivocal lesions reflects everyday clinical practice at our institution, where patients with a normal bone scintigraphy or harboring several obvious malignant lesions are not succumbed to additional SPECT/CT.

## Conclusion

^68^Ga-PSMA-11 PET/CT exhibited few equivocal bone findings and revealed bone metastases in 10% of patients with newly diagnosed PCa and negative BS results. However, PSMA-avid lesions in the ribs without other PSMA-avid lesions outside the prostate should be interpreted cautiously as they often represent non-prostatic tissue.

## Supplementary information

**Additional file 1: Table S1.** Best valuable comparator for bone metastases in 41 patients with at least one positive lesion demonstrated by any of the imaging modalities (BS or PSMA PET/CT).

## Data Availability

Please contact the corresponding author for data requests.
