# Supplementary material for: Added value of 68Ga-PSMA PET/CT for the detection of bone metastases in patients with newly diagnosed prostate cancer and a previous 99mTc bone scintigraphy
Source: EJNMMI Res. 2020 Apr 8;10:31. doi: 10.1186/s13550-020-00618-0 (PMC7142208; doi:10.1186/s13550-020-00618-0)
Supplement: Supplementary file 1 — Additional file 1: Table S1. Best valuable comparator for bone metastases in 41 patients with at least one positive lesion demonstrated by any of the imaging modalities (BS or PSMA PET/CT). [file 13550_2020_618_MOESM1_ESM.docx]

Supplementary table 1: Best valuable comparator for bone metastases in 41 patients with at least one positive lesion demonstrated by any of the imaging modalities (BS or PSMA PET/CT).

| **Patient** | **PSMA-PET/CT*** | **Bone scan** | **Additional Imaging before or after study related procedures** | **Interpretation of imaging and clinical follow up including PSA-values** | **BVC at the patient level** |
| --- | --- | --- | --- | --- | --- |
| 1 | M1;  *Th7 | M0 | Anatomical MRI at staging  2 ceCT at staging and at 30 months | The patient received radiotherapy of the prostate and ADT.  MRI probable hemangioma.  ceCT no morphologic changes during ADT compared to pretreatment.  No skeletal-related symptoms† | Me |
| 2 | Me;  *L5 | Me;  *8^th^ left rib | None | No skeletal-related symptoms†  ADT, with PSA stable (0.5 ng/mL) for more than 2 years | Me |
| 3 | M1;  *Left ischiadic bone | M1;  *Left ischiadic bone | 6 ceCT the latest at 20 months  3 MRI the latest at 20 months  5 x-ray the latest at 19 months | ceCT, MRI and X-ray confirm BM and with increasing number of BM  Bone related pain. Died from disseminated PCa | M1 |
| 4 | Me;  *5^th^ left rib | Me;  *5^th^ left rib | 2 ceCT at staging and at 26 months | No changes on ceCT during 26 months of ADT. CT-morphology benign.  No clinical symptoms indicating BM. Stable PSA at 2.5 ng/mL | M0 |
| 5 | M1;  *Left iliac bone | M1;  *Left iliac bone | 3 ceCT at staging, at 12 and 24 months | Increasing sclerosis of the metastases in os ilium after start of ADT  No skeletal-related symptoms† | M1 |
| 6 | M0 | Me;  *12^th^ right rib  *5^th^ left rib | PSMA PET/ceCT at 3 months | PSMA no BM, no sclerotic lesions in ribs after start of ADT | M0 |
| 7 | M0 | Me;  *S1 | NaF PET/CT  ceCT at staging | No BM on NaF PET/CT and ceCT,  RP with immeasurable PSA for 12 months (ongoing) | M0 |
| 8 | M0 | Me;  *4^th^ right rib | MRI at staging  ceCT at 12 months | ceCT no BM at follow up. No skeletal-related symptoms†  Have received radiotherapy combined with ADT. PSA immeasurable for more than a year (ongoing). | M0 |
| 9 | Me;  *Th9 | M0 | PSMA PET/CT at 3 months  MRI of pelvis and ceCT at staging | ceCT and PSMA PET/CT at follow up without BM.  Have received radiotherapy combined with ADT -> immeasurable PSA for more than a year (ongoing). No skeletal-related symptoms† | M0 |
| 10 | M1;  *Right pubic bone  *Left pubic bone | Me;  *Right pubic bone | ceCT at staging | ceCT confirms BM | M1 |
| 11 | M0 | Me;  *5^th^ left rib  *9^th^ right rib | None | No treatment, wait and watch, stable PSA for 18 months | M0 |
| 12 | M1;  *Left pubic bone | Me;  *Left pubic bone | ceCT at 1 month | ceCT confirms BM | M1 |
| 13 | M1;  *4 in left pubic/ischiadic bone  *5 in right pubic/ischiadic bone | M1  *3 in right pubic  Bone  *one in left pubic bone | 4 ceCT the latest at 14 months  2 BS, the latest at 14 months | ceCT and BS confirms BM and with increasing sclerosis after start of ADT | M1 |
| 14 | M0 | Me;  *Sacral bone | mpMRI of the pelvis at 1 month | mpMR shows no BM in the pelvis.  RP with immeasurable PSA for 12 months (ongoing) | M0 |
| 15 | M0 | Me;  *7^th^ right rib | ceCT | Normal bone morphology on ceCT. RP with PSA=0.2 ng/mL after 3 months, 0.3 at 6 months (eLND revealed LN metastases) | M0 |
| 16 | M1;  *Left clavicle, *Right iliac bone  *L3 | Me;  *Right iliac bone | 2 BS the latest at 12 months  3 ceCT the latest at 12 months | BS and ceCT confirms BM and progression, increasing sclerosis after start of ADT | M1 |
| 17 | M1;  *Th10  *Right pubic bone | M1;  *Th10  *Right pubic bone | MRI of the pelvis at 6 months  3 ceCT the latest at 15 months  2 PSMA-PET/CT the latest at 6 months | All imaging confirms the presence of BM and with increasing number of bone metastases  Increasing bone related pain, receives bone targeted radiotherapy of the right pubic bone, dies from PCa | M1 |
| 18 | M1;  >10 bone lesions | M1;  *Th1  *Th9  *9^th^ right rib | 4 BS the latest at 16 months  6 ceCT the latest at 18 months  PSMA-PET/CT at 3 months | All imaging confirms BM, with progression and increasing sclerosis after start of ADT  Operated for spinal cord compression (histopathology of removed bone showed metastases from PCa) | M1 |
| 19 | M1;  *Right femoral bone  *3 in pelvis  *11^th^ left rib | M1;  * Right femoral bone  *2 in pelvis *11^th^ left rib | 2 BS the latest at 13 months  2 ceCT the latest at 13 months | BS and ceCT confirms BM with increasing sclerosis after start of ADT, and osteolytic BM | M1 |
| 20 | M1;  *4^th^ right rib | Me;  4^th^ right rib  *2^nd^ left rib  *7^th^ left rib  *L1 | CT-guided biopsy of 4^th^ right rib | Biopsy from left 4^th^ rib: no BM, RP with immeasurable PSA for 12 months (ongoing) | M0 |
| 21 | M1;  *Sacral bone  *Right iliac bone | M1;  *Sacral bone  *Right iliac bone | MRI at 15 months | MRI confirms BM, plus additional new BM. Bone related pain | M1 |
| 22 | M0 | Me;  *7^th^ right rib | MRI at staging | MRI without BM. The patient undergoes radio therapy with ADT, PSA is unmeasurable for 14 months (ongoing). No skeletal-related symptoms† | M0 |
| 23 | M1,  *7^th^ left rib | M0 | CT 6 months prior to staging  ceCT at 14 months | CT without any morphologic changes, no new lesions or sclerosis after 14 months of ADT. No skeletal-related symptoms† | M0 |
| 24 | Me;  *7^th^ left rib | M0 | None | Treated with high-intensity focused ultrasound | Me |
| 25 | M1;  *2 in pelvis  *Left scapula | M0 | BS x 2, the latest at 13 months  ceCT at 6 months | BS and ceCT confirms increasing sclerosis of BM during ADT | M1 |
| 26 | M0 | Me;  *10^th^, 11^th^ right rib  *10^th^ left rib | None | RP with immeasurable PSA at 12 months (ongoing) | M0 |
| 27 | M1;  *4th right rib | M0 | None | ADT, no skeletal-related symptoms† | Me |
| 28 | M0 | Me;  Left acetabular region | FDG PET/CT 3 months | *No bone lesions. RP with immeasurable PSA AT 12 months (ongoing) | M0 |
| 29 | M1;  *6^th^ left rib | Me  *6^th^ left rib | None | RP with immeasurable PSA AT 6 months (ongoing) | M0 |
| 30 | M1;  *Right pubic bone  *9^th^ left rib. | Me;  *Right pubic bone  *9^th^ left rib. | ceCT at staging | ceCT shows distinct sclerotic lesions in the right pubic bone | M1 |
| 31 | M1;  *Right pubic bone  *Right sacroiliac joint  *Right iliac bone | M1;  *Right pubic bone  *Right sacroiliac joint  *Right iliac bone | ceCT at staging | ceCT shows distinct sclerotic lesions corresponding to the findings in PSMA PET/CT and BS | M1 |
| 32 | M0 | Me  *9^th^ left rib | ceCT at staging and at 10 months | ceCT without any suspicious lesions. Wait and watch with stable PSA for 12 months (ongoing). No skeletal-related symptoms† | M0 |
| 33 | M1;  *Right pubic bone (two) | M0 | None | ADT | Me |
| 34 | M1;  *Left iliac bone | Me;  *Right scapula  *8^th^ left rib | ceCT 5 years prior to staging, at staging and at 12 months  BS 10 months | CT without any lesions 5 y prior to diagnosis. Sclerotic lesion in iliac bone at staging and with increasing sclerosis during ADT  BS no metabolic active lesions during follow up G-RAMPP (RP+ ADT) PSA at 12 months <0.1 | M1 |
| 35 | M1;  *7^th^ right rib  *8^th^ left rib | M0 | None | RP with immeasurable PSA at 10 months (ongoing) | M0 |
| 36 | M1;  *Left scapula | Me;  *7^th^ left rib, *Left scapula | ceCT 10 months prior to staging and at staging | ceCT without morphologic changes over time, no certain malignant lesions | Me |
| 37 | M0 | Me;  *L1 | ceCT at staging | No lesions on ceCT  Radiotherapy and 3 years of ADT with immeasurable PSA at 12 months (ongoing) | M0 |
| 38 | M1;  *Right pubic bone | M1;  *Right pubic bone | ceCT at staging | ceCT with distinct sclerotic lesion | M1 |
| 39 | M1;  *Th2  *Th5  *Th9  *Th11  *Right iliac bone  *Left arm  *5^th^ right rib  *7^th^ left rib | M1;  *Th9  *Left arm  *5^th^ right rib  *7^th^ left rib | ceCT at staging | ceCT with distinct sclerotic lesions | M1 |
| 40 | M1;  *Th8  *Left iliac bone  *Right pubic bone | M0 | ceCT at staging | ceCT with distinct sclerotic lesion in the left iliac bone and a slight sclerotic lesion in Th8 | M1 |
| 41 | M1;  *L3  *9^th^ left rib | M0 | None | ADT, no skeletal-related symptoms† | Me |

* Consensus diagnosis of independent observers, † Skeletal-related symptoms are defined as bone pain and/or the need for bone-targeted radiotherapy, fractures or spinal cord compression.

M0: No bone metastases, Me: Equivocal for bone metastases, M1: Bone metastases, BVC: Best valuable comparator, BM: Bone metastases, LN: Lymph nodes, ceCT: Contrast-enhanced CT, BS: Bone scintigraphy, RP: Radical prostatectomy, ADT: Androgen deprivation therapy, eLND: extended lymph node dissection.
